# Unraveling the Key Odorants in Floral-Fruity Aroma Pu-Erh Tea via GC-O-MS, GC-MS/MS, Aroma Recombination, and Omission Tests

**DOI:** 10.3390/foods14183223

**Published:** 2025-09-17

**Authors:** Xianxiu Zhou, Jiajing Hu, Hongchun Cui, Jiahao Tang, Yongwen Jiang, Haibo Yuan, Jiahua Li, Yanqin Yang

**Affiliations:** 1 College of Tea Science, Yunnan Agricultural University, Kunming 650201, China; 2 State Key Laboratory of Tea Plant Germplasm Innovation and Resource Utilization, Tea Research Institute, Chinese Academy of Agricultural Sciences, Hangzhou 310008, China; 3 Tea Research Institute, Hangzhou Academy of Agriculture, Hangzhou 310024, China

**Keywords:** floral–fruity aroma Pu-erh tea, GC-MS/MS, GC-O-MS, aroma recombination, omission test

## Abstract

Floral–fruity aroma Pu-erh tea (FFAPET) is highly valued by consumers for its distinctive and appealing fragrance. This study conducted a comprehensive investigation into the volatile components of FFAPET using advanced analytical techniques, including GC-O-MS, GC-MS/MS, aroma recombination, and omission tests. Compared with traditional stale aroma Pu-erh tea, FFAPET exhibited higher levels of alcohols and alkenes. Through GC-O-MS combined with OAV analysis, 10 key active-aroma compounds were identified in representative FFAPET samples. These compounds included (*E, E*)-2,4-heptadienal, phenylethyl alcohol, geraniol, (*E*, *Z*)-2,6-nonadienal, (*Z*)-4-heptenal, 2-phenethyl acetate, 2,2,6-trimethylcyclohexanone, *D*-limonene, *β*-ionone, and linalool, all of which made significant contributions to the development of the characteristic floral–fruity aroma profile. Tests involving aroma recombination and omission further confirmed that seven of these odorants played essential roles in defining the overall aroma of FFAPET. These findings offer valuable theoretical insights for the enhancement and regulation of Pu-erh tea flavor, particularly for the optimization of the floral–fruity aroma characteristics.

## 1. Introduction

Pu-erh tea, originating from Yunnan Province in southwestern China, is recognized as a microbial post-fermented tea [1]. The production process involves placing the humidified raw materials under high temperature and high humidity for a specific period until the tea leaves turn reddish-brown and the astringency disappears, after which they are dried. Pu-erh tea is renowned for its distinctive aroma and numerous health benefits, including antioxidant, anti-tumor, and hypolipidemic properties, contributing to its widespread popularity among consumers [2,3]. Traditionally, Pu-erh tea is characterized by a stale aroma. In recent years, special variants with unique aromatic profiles—such as floral–fruity aroma—have emerged to meet consumer demand for diversified flavors. These innovative products not only enrich the diversity of Pu-erh tea offerings but also attract new consumer groups, thereby enhancing the market competitiveness and cultural appeal of Pu-erh tea. Nevertheless, systematic studies on the key odorants in floral–fruity aroma Pu-erh tea (FFAPET) are still lacking, which severely restricts the standardization and quality control of such specialty teas. Identifying the volatile compounds responsible for the floral–fruity aroma is essential for optimizing production processes, ensuring aroma consistency, meeting consumer expectations, and promoting the sustainable development of the Pu-erh tea industry.

Aroma is a crucial factor for assessing tea quality, despite accounting for only 0.005% to 0.02% of the total chemical constituents [4]. To date, over 700 volatile components have been identified in tea. Variations in their composition and concentration contribute significantly to the diversity of tea aroma profiles. Nevertheless, a small subset of these aroma components plays an essential role in defining the overall sensory characteristics of tea. Sensomics has emerged as a powerful analytical strategy in flavor chemistry, revolutionizing the field of aroma research by providing a comprehensive and systematic approach to understanding complex flavor profiles. This innovative methodology integrates advanced analytical techniques with sensory evaluation to identify, characterize, and quantify key flavor-active compounds in food and beverages [5,6]. At its core, Sensomics combines instrumental analysis, such as gas chromatography-mass spectrometry (GC-MS), gas chromatography-olfactometry (GC-O), and aroma extract dilution analysis (AEDA), with sensory-driven approaches like odor activity value (OAV) determination and aroma recombination experiments. This multi-dimensional strategy enables researchers to bridge the gap between chemical composition and sensory perception, offering unprecedented insights into the molecular basis of flavor [7]. It has been successfully applied in various tea aroma studies, including the characterization of fungal-aroma components in Fu Brick tea and the identification of essential aroma components in Yunnan black tea [4,8].

In the present study, a comprehensive sensomics-based strategy was systematically employed to identify and characterize the key odorants responsible for FFAPET. The approach integrated advanced analytical techniques, including gas chromatography–tandem mass spectrometry (GC-MS/MS), OAV, GC-O-MS, and AEDA. To validate the individual contributions of identified odorants in shaping the characteristic floral–fruity aroma, subsequent experiments involving aroma recombination and omission tests were conducted. The findings are expected to establish a robust scientific framework for targeted flavor modulation and quality optimization in Pu-erh tea production.

## 2. Materials and Methods

### 2.1. Chemicals and Materials

A total of 18 ripened Pu-erh tea samples were gathered from Pu-erh tea production enterprises in Yunnan, China. Based on comprehensive sensory assessments performed by certified tea evaluation experts, these samples were categorized into two distinct groups: 10 samples exhibiting a floral–fruity aroma (designated as FFAPET1-FFAPET10) and 8 samples characterized by a stale aroma (designated as SAPET1-SAPET8). A DVB/CAR/PDMS fiber (50/30 μm, 2 cm; Supelco, Bellefonte, PA, USA) with a manual holder and specialized headspace vials (20 mL; Agilent Technologies, Santa Clara, CA, USA) were used for volatile extraction. Ethyl decanoate was supplied by Aladdin Industrial Corporation (Shanghai, China), and purified water was purchased from Wahaha Group Co., Ltd. (Hangzhou, China). The detailed information regarding the standards of volatile compounds is provided in Table S1.

### 2.2. GC-MS/MS Analysis

Volatile compounds in 18 Pu-erh tea samples were extracted using headspace solid-phase microextraction (HS-SPME) following previously established methods [9]. A DVB/CAR/PDMS fiber was employed due to its broad sensitivity and strong affinity for a wide range of volatile and semi-volatile compounds. The preparation of each sample involved homogenizing tea samples (0.5 g) with pure water (5 mL) into headspace vial, supplemented with ethyl decanoate (5 μL, 100 mg/L) as an internal standard. The fiber was subsequently subjected to exposure within the headspace of the vial to facilitate the adsorption of volatile compounds. This process was followed by an incubation period at a temperature of 60 °C for a duration of 60 min. After the incubation, desorption occurred at 270 °C for 5 min to release the adsorbed analytes.

Volatile compounds were analyzed using an Agilent 7890B/7000C GC-MS/MS system equipped with a DB-5MS capillary column (30 m × 0.25 mm × 0.25 μm) to achieve optimal separation. High-purity helium (99.999%) was used as the carrier gas at a constant flow rate of 1.0 mL/min under splitless injection mode. The temperature protocol commenced at 40 °C and was maintained for a period of 5 min. Subsequently, the temperature was incrementally raised to 160 °C at 4 °C/min (holding for 2 min). After that, it was further increased to 270 °C at 10 °C/min (holding for 2 min). MS parameters were as follows: electron ionization mode (70 eV), mass range of 40–450 *m*/*z*, ion source temperature of 230 °C, and transfer line temperature of 250 °C.

The volatile components were qualitatively analyzed using a dual-validation approach: (1) mass spectral matching against the NIST11 library (similarity > 80%), and (2) retention index (RI) verification via a C7–C40 n-alkane calibration. The RI values were cross-referenced with authoritative databases, such as the NIST Chemistry WebBook (https://webbook.nist.gov/chemistry/, accessed on 28 August 2025). Additionally, some key aroma compounds were further verified by authentic chemical standards. Quantification was performed using the external standard method as described in our previous work [9], with the specific standard curves detailed in Table S2. For compounds lacking commercial standards, concentrations were determined using a semi-quantitative approach using ethyl decanoate as the internal standard.

### 2.3. Odor Activity Value Analysis

The OAV is frequently used to evaluate the contributions of aroma substances. It is widely recognized that aroma substances with OAVs > 1 are considered significant contributors to aroma, as their concentrations surpass sensory detection thresholds and thus play a crucial role in shaping the perceived olfactory profile. The OAV is calculated according to the formula OAV = C*_i_*/OT*_i_*, where C*_i_* represents the measured concentration of a specific volatile compound, and OT*_i_* corresponds to its odor threshold value in water.

### 2.4. Gas Chromatography-Olfactometry-Mass Spectrometry Analysis

The GC-O-MS analysis was conducted on a representative floral–fruity Pu-erh tea named FFAPET2, using an Agilent 7890A/5975C GC-MS system coupled to an olfactory detector (ODP4, Heidelberg, Germany). Separation was achieved using a HP-Innowax capillary column (60 m × 0.25 mm × 0.25 μm). The GC temperature program was set as follows: 40 °C (holding for 2 min) → 135 °C (ramping at 6 °C/min) → 240 °C (ramping at 4 °C/min) → 250 °C (ramping at 10 °C/min, and holding for 5 min). The column effluent was split equally, with half directed to the MS detector and the other half to the sniffing port. Six assessors (3 males and 3 females) independently conducted the sniffing tests, recording detailed descriptors of aroma characteristics along with their corresponding intensities. The AEDA technique was carried out according to the method established by Feng et al. [10]. Briefly, serial dilutions were achieved by progressively increasing the split ratio. The flavor dilution (FD) factor was systematically evaluated at dilution ratios of 1:1, 1:3, 1:7, 1:15, 1:31, 1:63, and 1:127, with each dilution level analyzed in triplicate to ensure reproducibility.

### 2.5. Aroma Recombination and Omission Tests

Volatile-free samples were prepared by adding tea samples (50 g) and distilled water (150 mL) in a round-bottom flask, followed by rotary evaporation to remove the aroma compounds. The samples were then rehydrated repeatedly at least six times at 40 °C to ensure the complete removal of residual aroma components. Finally, the volatile-free matrix samples were freeze-dried for subsequent analysis. Ethical permission was not required for the sensory study, and all participants provided informed consent to participate in the sensory evaluation.

In the aroma recombination test, volatile compounds in FFAPET with OAVs exceeding 1 and simultaneously sniffed by GC-O-MS were incorporated into the tea infusion prepared from the volatile-free tea samples at their naturally occurring concentrations. Subsequently, a quantitative descriptive analysis (QDA) was conducted by 10 trained judges (five females and five males) with over three years of experience and senior appraiser qualifications. Through discussion and evaluation, 6 distinct odor attributes were established, namely fruity-floral, stale, green, fatty, medicinal, and woody [11]. Sensory intensities were rated on a 10-point scale, where “1” represented barely perceivable, “5” represented moderate intensity, and “10” a represented strong intensity.

The aroma omission experiments were conducted following an established approach [4]. The experimental models were prepared: one omission model (lacking a target odorant) and two complete models (containing all aroma compounds), followed by a random code with three digits. The sensory evaluation team sniffed the samples to identify those that differed from the complete model. Significant differences were analyzed using the reported methodology [12].

### 2.6. Data Analysis

All experiments were conducted in triplicate to ensure reproducibility. Statistical significance was assessed using SPSS 20.0 (IBM, Armonk, NY, USA). Multivariate statistical analysis was performed using SIMCA 14.1 (Umetrics, Umeå, Sweden). Pie charts and radar charts were generated using Origin 2021 (OriginLab, Northampton, MA, USA), while box plots and heatmaps were created by Chiplot and TBtool, respectively.

## 3. Results and Discussion

### 3.1. Comparative Analysis of the Volatile Profiles in FFAPET Samples with SAPET Samples via GC-MS/MS

#### 3.1.1. Characterization of the Volatile Compounds in FFAPET Samples as Compared with SAPET Samples

**Figure 1:**
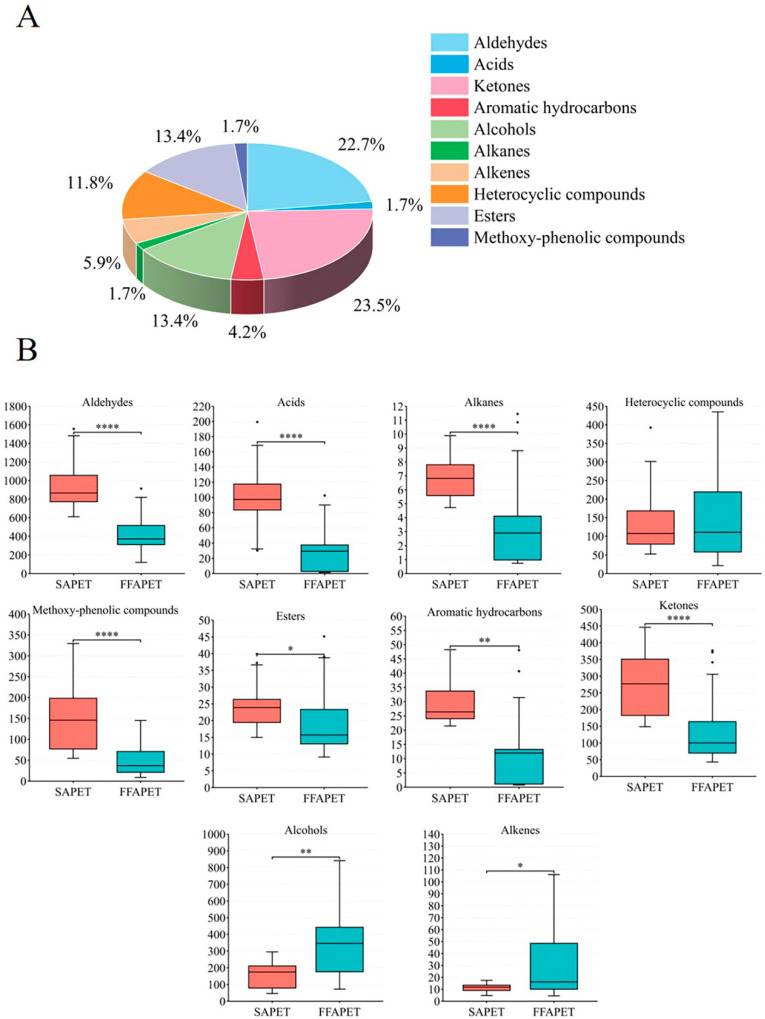
Comparison of volatile compounds in FFAPET and SAPET samples. (**A**) The proportions of different types of volatile compounds; (**B**) Content comparisons of different categories of volatile compounds. FFAPET represented floral–fruity aroma Pu-erh tea; SAPET represented stale aroma Pu-erh tea. Statistical significance was determined as follows: * (*p* ≤ 0.05), ** (*p* ≤ 0.01), and **** (*p* ≤ 0.0001).

The volatile components in FFAPET and SAPET samples were systematically analyzed using GC-MS/MS. In total, 119 volatile compounds were successfully identified through database searching, retention indices, and confirmation with authentic standards (Tables S3 and S4). Based on their chemical structure, these volatiles were classified into 10 subcategories: 27 aldehydes, 2 acids, 28 ketones, 5 aromatic hydrocarbons, 16 alcohols, 2 alkanes, 7 alkenes, 14 heterocyclic compounds, 16 esters, and 2 methoxy-phenolic compounds. As shown in Figure 1A, ketones accounted for the highest proportion (23.5%) in terms of quantity, followed by aldehydes (22.7%), alcohols (13.4%), and esters (13.4%). The content levels of alcohols and alkenes in the FFAPET samples ranged from 72.38 to 1436.01 μg/L and from 4.47 to 68.44 μg/L, respectively, which were significantly higher than those in the SAPET samples (*p* < 0.05) (Figure 1B). Alcohols, which generally impart pleasant floral and fruity aromas, significantly contribute to the distinctive flavor profile of tea [8]. The total contents of volatile compounds including aldehydes (120.11~912.90 μg/L), acids (1.53~62.21 μg/L), ketones (43.30~376.76 μg/L), aromatic hydrocarbons (0.78~73.69 μg/L), alkanes (0.74~14.26 μg/L), and methoxy-phenolic compounds (8.84~145.43 μg/L), were significantly lower in FFAPET samples compared to SAPET (*p* < 0.05). Methoxy-phenolic compounds are a unique class of compounds that distinguish Pu-erh tea from other types of tea, and are integral to the characteristic “stale” aroma [13].

#### 3.1.2. Multivariate Statistical Analysis

**Figure 2:**
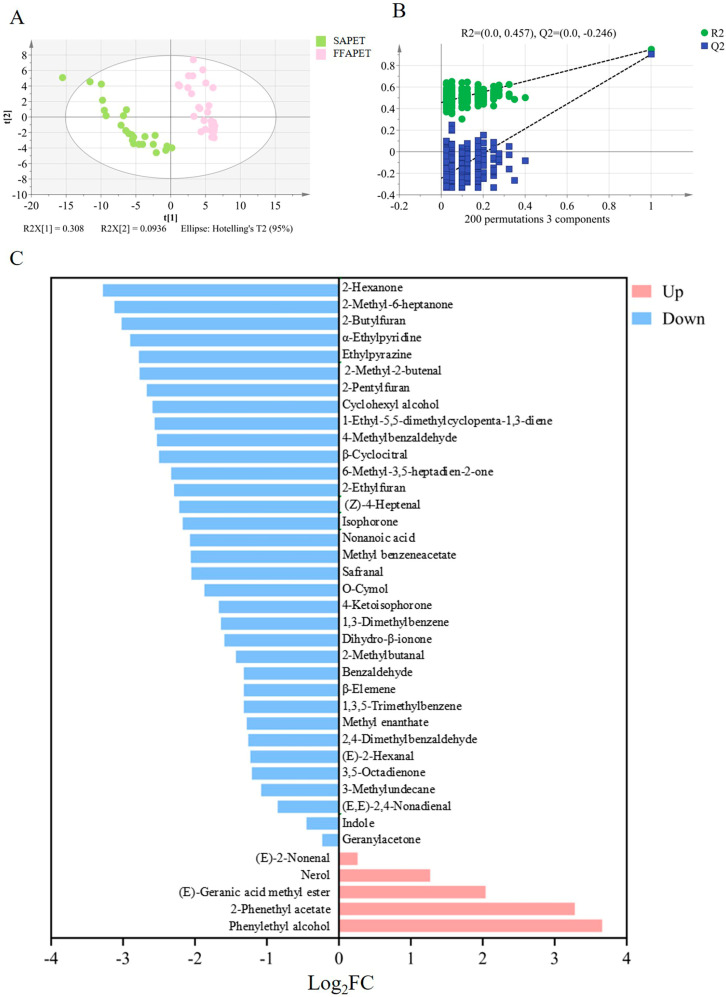
Multivariate statistical analysis of volatile compounds in FFAPET and SAPET samples. (**A**) The score plots of PLS-DA; (**B**) Permutation tests with 200 iterations; (**C**) Significant alterations in aroma compounds. FFAPET represented floral–fruity aroma Pu-erh tea; SAPET represented stale aroma Pu-erh tea.

To clarify the disparities between FFAPET and SAPET samples and identify the key differential volatile substances, PLS-DA was employed to conduct a comprehensive analysis of the 119 volatile components. As illustrated in Figure 2A, a distinct separation between FFAPET and SAPET samples was observed in the PLS-DA score plots, with FFAPET samples positioned on the positive *X*-axis and SAPET samples on the negative *X*-axis. The model demonstrated strong explanatory power and predictive capability, as indicated by the parameters R^2^Y = 0.947 and Q^2^ = 0.903. Additionally, permutation tests with 200 iterations (R^2^Y = 0.457, Q^2^ = −0.246) confirmed the reliability of the model (Figure 2B).

Typically, the variable important on projection (VIP) is used to assess the impact of individual variables on classification, with VIP values greater than 1 considered significant [14]. In this study, 52 key volatile compounds with VIP > 1 were identified (Figure S1). By integrating one-way analysis of variance, 39 key differential volatiles were screened out based on the criteria of *p* < 0.05 and VIP > 1. The fold changes (FC) of these 39 aroma compounds were illustrated in Figure 2C. Among these, 5 volatile compounds were upregulated (Log_2_FC > 0), while 34 volatile compounds were downregulated (Log_2_FC < 0). Notably, compounds with Log_2_FC > 1, including phenylethyl alcohol (floral, rose), 2-phenethyl acetate (floral, rose), (*E*)-geranic acid methyl ester (floral, orange), and nerol (floral, green, sweet, lemon-like), were significantly higher in FFAPET compared to SAPET. These volatiles are crucial in contributing to the floral and fruity scents characteristic of Pu-erh tea. These differences can be attributed to variations in tea varieties, growing environments, and processing techniques [15].

### 3.2. Screening the Key Odorants in FFAPET by a Combination of OAV and GC-O-MS Analysis

#### 3.2.1. OAV Analysis of Key Volatile Compounds

**Figure 3:**
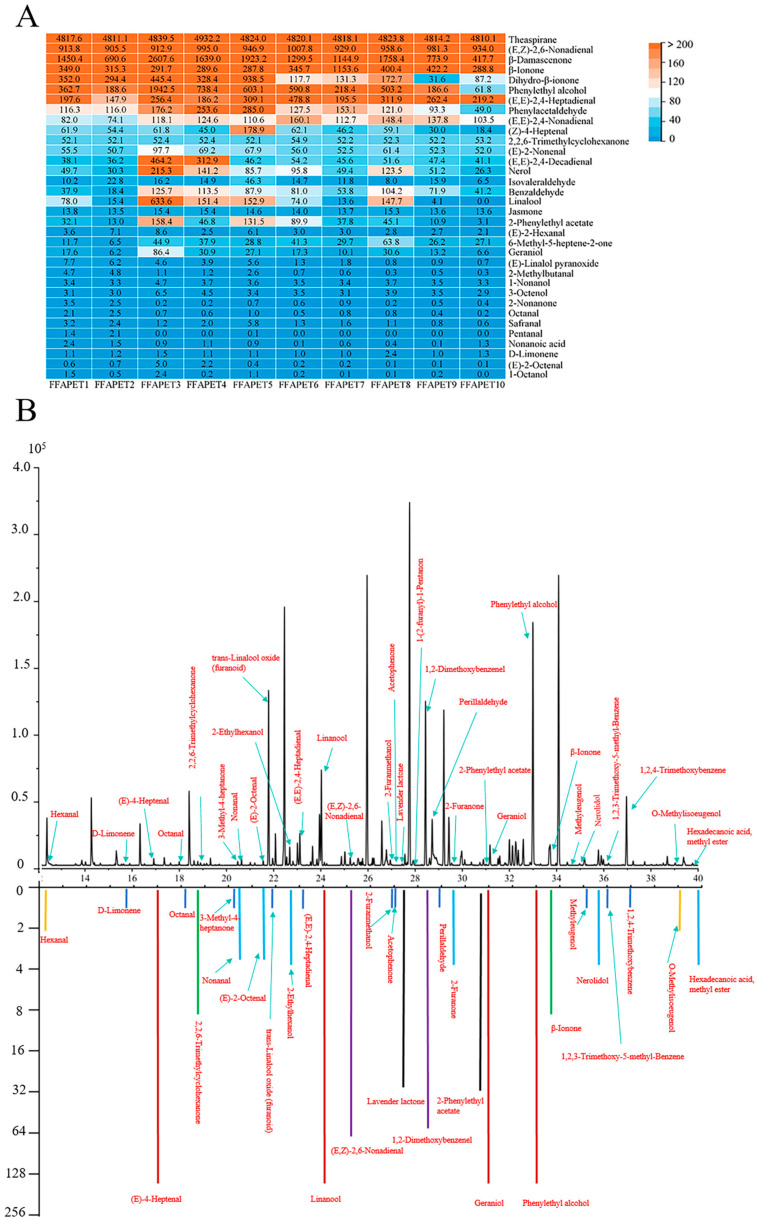
Identification of key volatile compounds. (**A**) Heat map of 34 key volatile compounds with OAVs ≥ 1; (**B**) Total ion chromatograms and aroma extract dilution analysis of representative FFAPET2. FFAPET represented floral–fruity aroma Pu-erh tea.

The aromatic characteristics of tea are shaped by intricate interactions among a variety of aroma compounds, resulting in distinct aroma types [15]. Compounds with OAV greater than 1 are typically regarded as significant contributors to the overall aroma profile [16]. Moreover, compounds with higher OAVs exert a more pronounced influence on the overall fragrance. In total, 34 active-aroma compounds were identified in FFAPET samples using OAV analysis. The OAVs for these compounds are presented in Table S5, while their respective distributions are illustrated in Figure 3A. The color-coded squares represent the variations in OAVs: blue squares indicate lower OAVs, while orange squares signify higher OAVs. Specifically, theaspirane (OAV = 4810.1~4932.2), (*E*, *Z*)-2,6-nonadienal (OAV = 905.5~1007.8), *β*-damascenone (OAV = 417.7~2607.6), *β*-ionone (OAV = 287.8~1153.6), dihydro-*β*-ionone (OAV = 31.6~938.5), phenylethyl alcohol (OAV = 61.8~1942.5), (*E*, *E*)-2,4-heptadienal (OAV = 147.9~478.8), benzeneacetaldehyde (OAV = 49.1~285), (*E*, *E*)-2,4-nonadienal (OAV = 74.1~160.1), (*Z*)-4-heptenal (OAV = 18.4~178.9), 2,6,6-trimethylcyclohexanone (OAV = 52.1~54.9) exhibited higher OAVs, indicating these compounds potentially contributed to the aroma profile of FFAPET samples.

*β*-Damascenone and *β*-ionone are known for their pleasant floral fragrances [17]. Previous studies have suggested that these compounds may exhibit a synergistic effect in enhancing floral properties, underscoring their significant contribution to the distinctive scent of FFAPET samples [14]. Theaspirane is widely present in various foods and beverages such as tea, wine, and grapes, and is characterized by a woody and refreshing odor profile [18,19]. Even at low concentrations, theaspirane significantly enhances the aroma profile of green tea [19]. In summary, compounds with high OAVs are crucial in defining the characteristic floral–fruity aroma of FFAPET samples.

#### 3.2.2. GC-O-MS Analysis

To identify the odor-active constituents that contribute to the distinctive characteristics of FFAPET, GC-O-MS in conjunction AEDA was conducted on the representative FFAPET sample (FFAPET2). As shown in Table S6 and Figure 3B, a total of 31 odor-active compounds were successfully identified through GC-O-MS analysis, including 9 alcohols, 4 methoxy-phenolic compounds, 8 aldehydes, 6 ketones, 1 alkene, and 3 esters. Among them, linalool, geraniol, phenylethyl alcohol, and (*Z*)-4-heptenal exhibited the highest FD factors (FD = 128), indicating their essential role as key aroma substances in FFAPET. In addition, 6 aroma-active compounds such as (*E*, *Z*)-2,6-nonadienal (FD = 64), 2-methylacetophenone (FD = 32), 2-phenylethyl acetate (FD = 32), lavender lactone (FD = 16), *β*-ionone (FD = 8), and 2,2,6-trimethylcyclohexanone (FD = 8) were identified as significant contributors to the floral–fruity odors of FFAPET samples. These compounds are characterized by cheerful floral, fruity, and sweet aroma characteristics. Lavender lactone has been identified as a natural constituent in honey and is regarded as an organoleptically intriguing compound [20]. It is an oxidative metabolite derived from linalool. The generally accepted pathway involves the oxidation and cyclization of linalool to form furanoid linalool oxide, which subsequently undergoes dehydration to yield dehydrolinalool oxide. This intermediate is further transformed through oxidation and lactonization to produce lavender lactone [21]. Additionally, compounds such as 1-hexanol, nerolidol, nonanal, (*E*)-2-octenal, and methyl hexadecanoate exhibited FD factors of 4. Although their FD factors were lower than those of the aforementioned substances, they still contributed to the development of the aromatic profile of FFAPET.

#### 3.2.3. Screening the Key Odorants Responsible for FFAPET

As indicated by the results, a notable divergence was observed between the aroma-active compounds identified by OAV and those detected by GC-O-MS. This discrepancy may be attributed to variations in reported odor thresholds across different literature sources, as well as the inherent subjectivity associated with human olfactory assessment during GC-O-MS analysis [22]. By integrating these two complementary methods, it was possible to overcome the limitations specific to each technique. Based on the consensus compounds identified through both approaches, 10 key volatile compounds—namely (*E*, *E*)-2,4-heptadienal, (*E*, *Z*)-2,6-nonadienal, (*Z*)-4-heptenal, phenylethyl alcohol, 2-phenethyl acetate, 2,2,6-trimethylcyclohexanone, *β*-ionone, *D*-limonene, geraniol, and linalool—were identified as key odorants responsible for FFAPET.

**Figure 4:**
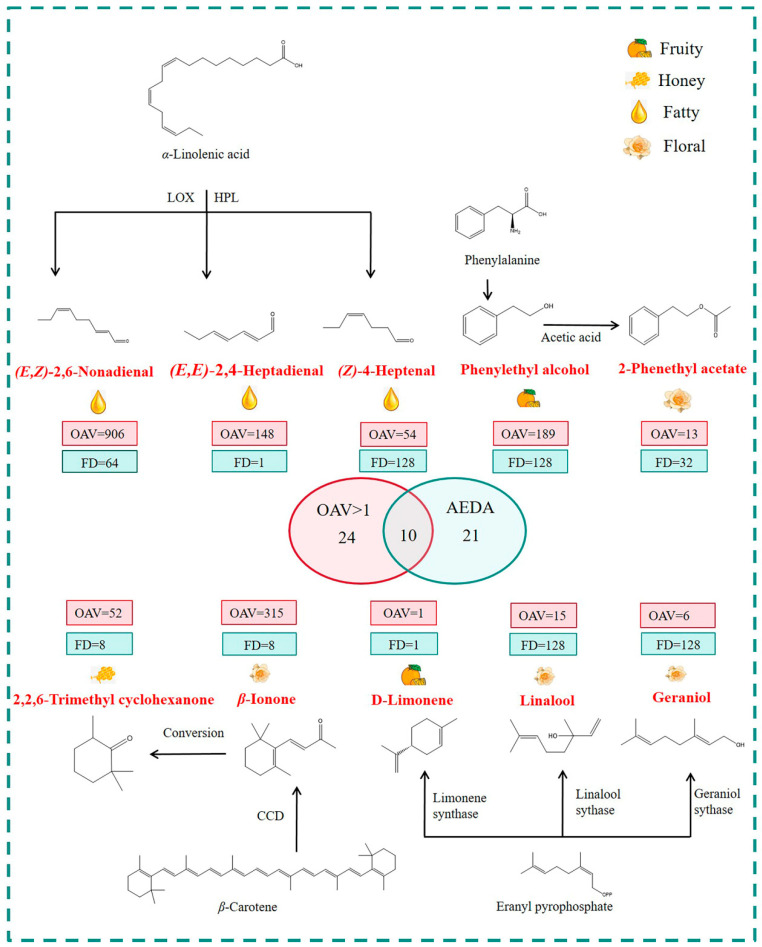
A total of key odorants in representative FFAPET2. FFAPET represented floral–fruity aroma Pu-erh tea.

As illustrated in Figure 4, representative compounds including linalool, geraniol, *β*-ionone, 2-phenylethyl acetate, and phenylethyl alcohol played a key role in defining the distinctive aroma profile. Among these, phenylethyl alcohol and 2-phenethyl acetate are generated from phenylalanine through the Strecker degradation pathway [23,24]. Notably, 2,2,6-trimethylcyclohexanone (FD = 8, OAV = 52) and *β*-ionone (FD = 8, OAV = 315) were identified as essential aroma compounds due to their highest FD factors and notable OAVs in FFAPET. These compounds are known to originate from the degradation of *β-*carotene. Additionally, *D*-limonene, along with geraniol and linalool, are derived from the common precursor geranyl pyrophosphate (geranyl- PP) through the enzymatic actions of limonene synthase, geraniol synthase, and linalool synthase, respectively [25]. Compounds such as (*E*, *Z*)-2,6-nonadienal (FD = 64, OAV = 906), (*E*, *E*)-2,4-heptadienal (FD = 1, OAV = 148), and (*Z*)-4-heptenal (FD = 128, OAV = 54) contributed fatty notes. These aldehydes are primarily generated through the oxidation of unsaturated fatty acids during tea processing, with the oxidation rate being directly proportional to their degree of unsaturation [26,27].

### 3.3. Verification of Key Odorants via Aroma Recombination and Omission Tests

#### 3.3.1. Aroma Recombination Experiment

**Figure 5:**
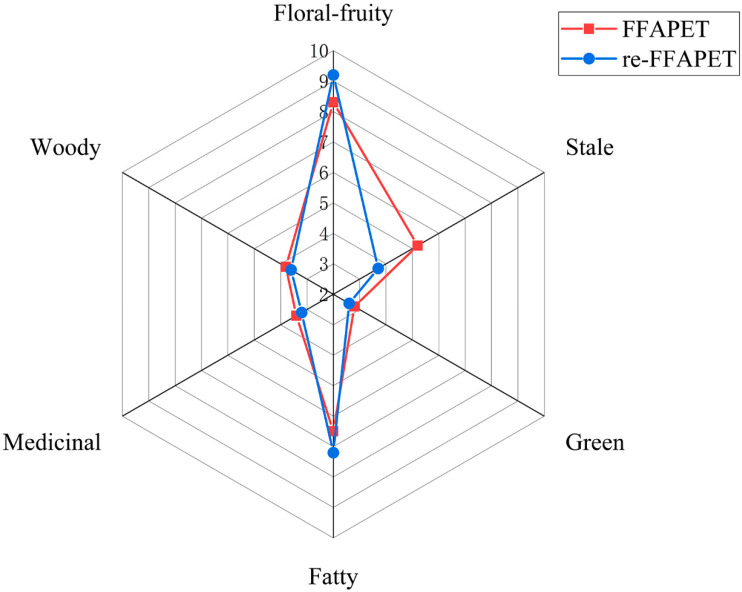
Aroma recombination of floral–fruity aroma profile versus the reconstituted floral–fruity aroma profile. FFAPET represented floral–fruity aroma Pu-erh tea; re-FFAPET represented reconstituted floral–fruity aroma Pu-erh tea.

Accordingly, 10 key odorants were incorporated into the volatile-free matrix at their natural occurring levels to prepare a recombinant model, termed *re*-FFAPET. QDA was conducted to evaluate six distinct odor attributes: stale, fruity-floral, green, fatty, medicinal, and woody. The results indicated that the aroma profile of the re-FFAPET closely resembled that of the original FFAPET model, with four attributes (green, medicinal, fatty, and woody) being nearly identical. However, slight differences were observed in the “floral–fruity” and “stale” attributes (Figure 5). Specifically, the re-FFAPET model scored lower in the stale aroma attribute compared to the original FFAPET sample. This could be attributed to the absence of certain synergistic interactions or background compounds in the recombinant model that might contribute to the stale note in the original sample. Conversely, the re-FFAPET model exhibited a more intense floral–fruity aroma compared to the original FFAPET sample. The interactions between non-volatile compounds and volatile components may explain this phenomenon. For instance, *β*-glucose can react with linalool to produce *β*-glucosides [27]. As time goes by, the glycosidic bonds can undergo hydrolysis under suitable conditions, slowly releasing aromatic compounds [28]. In contrast, the externally added linalool in the re-FFAPET model is free and thus releases its floral note more quickly, resulting in a stronger fruity-floral flavor. Overall, the recombinant model successfully simulated the typical aroma profile of FFAPET, confirming the critical role of these odorants in reproducing the characteristic floral–fruity aroma.

#### 3.3.2. Aroma Omission Experiment

Previous studies have indicated that the removal or reduction in specific odorants can markedly alter overall aroma profiles, largely due to synergistic, masking, or antagonistic interactions among volatile compounds [29]. To assess the contribution of individual odorants, aroma omission experiments were carried out, with the significance levels determined through triangulation tests [8]. The results revealed that three omission models showed no significant differences (*α* > 0.05), one omission model showed a significant difference (*α* ≤ 0.05), and six omission models displayed highly significant differences (*α* ≤ 0.01), as detailed in Table 1. Notably, the omission of (*E*, *Z*)-2,6-nonadienal was correctly identified by 70% of the evaluators (7 panelists, *α* ≤ 0.01), highlighting its critical role in the characteristic aroma of FFAPET. Additionally, the absence of *β*-ionone, linalool, and 2-phenethyl acetate was detected by 8 evaluators (*α* ≤ 0.01), while the omission of phenylethyl alcohol and geraniol was recognized by 7 evaluators (*α* ≤ 0.01), underscoring their substantial contributions to the floral–fruity aroma. These findings are consistent with GC-O-MS results (FD ≥ 8). In contrast, the omission of D-limonene resulted in a less perceptible change compared to key compounds such as (*E*, *Z*)-2,6-nonadienal and linalool, likely due to its relatively low level of FD. In addition, the omission of (*E*, *E*)-2,4-heptadienal, (*Z*)-4-heptenal, and 2,6,6-trimethylcyclohexanone did not yield significant changes in the aroma profile (*α* > 0.05), suggesting their minimal influence on the overall sensory characteristics.

In conclusion, compounds with higher OAVs and FD factors, such as linalool, geraniol, and 2-phenylethyl acetate, played a significant role in shaping the floral–fruity aromas of FFAPET. These compounds also made positive contributions to the flavor qualities observed during the aroma omission experiment. However, not all volatiles with high OAV or high FD factor (e.g., (*E, E*)-2,4-heptadienal and (*Z*)-4-heptenal) contributed significantly to the perceived aroma. This phenomenon may be attributed to interactive effects such as masking, antagonism, or synergy within the odorant mixture. Additionally, differences in the composition of the odorless matrix compared to the original tea matrix may also affect the reactivity and volatility of certain aroma compounds [10].

## 4. Conclusions

This study conducted a comprehensive analysis of the volatile components of FFAPET using GC-O-MS, GC-MS/MS, aroma recombination, and omission tests. Compared with SAPET, FFAPET exhibited higher levels of alcohols and alkenes. A significant difference between FFAPET and SAPET samples were achieved by PLS-DA, with high model robustness (R^2^Y = 0.947, Q^2^ = 0.903). A total of 31 active-aroma compounds were identified in representative FFAPET through GC-O-MS in conjunction with AEDA. By combining with OAV analysis, 10 key odorants including phenylethyl alcohol, (*E*, *E*)-2,4-heptadienal, 2-phenethyl acetate, (*Z*)-4-heptenal, (*E*, *Z*)-2,6-nonadienal, 2,2,6-trimethylcyclohexanone, *β*-ionone, *D*-limonene, geraniol, and linalool were screened out, which collectively contribute the floral and fruity aromas. Aroma recombination and omission tests further confirmed that seven of these odorants significantly contributed towards the development of the floral–fruity aroma profile. These findings provide valuable theoretical guidance for targeted flavor enhancement and regulation of Pu-erh tea, particularly for enhancing its floral–fruity aroma characteristics.

## Figures and Tables

**Table 1:** Significance level of each omission group in the aroma omission test.

No.	Compounds Omitted from Recombination Models	Odor Descriptions	n ^a^	Significance ^b^
1	(*Z*)-4-Heptenal	Fatty	3	n.s.
2	(*E*, *E*)-2,4-Heptadienal	Fatty, green, oily	5	n.s.
3	(*E*, *Z*)-2,6-Nonadienal	Fatty, green,	7	**
4	*β*-Ionone	Floral, violet-like	8	**
5	Linalool	Floral, rose	8	**
6	Phenylethyl alcohol	Floral, rose	7	**
7	Geraniol	Floral, rose	7	**
8	*D*-Limonene	Fruity	6	*
9	2-Phenethyl acetate	Floral, rose	8	**
10	2,6,6-Trimethylcyclohexanone	Sweet, honey-like	2	n.s.

^a^ The number of correct judgments from 10 sensory evaluators evaluating the aroma difference according to a triangle test; ^b^ significance: ** (*α* ≤ 0.01, high significant); * (*α* ≤ 0.05, significant); n.s. = not significant.

## Data Availability

The original contributions presented in this study are included in the article/Supplementary Materials. Further inquiries can be directed to the corresponding authors.

## Supplementary Materials

The following supporting information can be downloaded at: https://www.mdpi.com/article/10.3390/foods14183223/s1, Figure S1: Key differential volatile compounds with VIP >1; Table S1: The standard information of volatile compounds used in this study; Table S2: The information of qualitative and quantitative ion pairs and calibration curves; Table S3: The information of identified volatile compounds in SAPET and FFAPET samples; Table S4: The odor thresholds of volatile compounds; Table S5: A total of 34 volatile components with OAV > 1 in floral–fruity aroma Pu-erh tea samples; Table S6: The information of volatile compounds identified by GC-O-MS. References [30,31,32,33] are cited in the Supplementary Materials.
