# Supplementary material for: Unraveling the Key Odorants in Floral-Fruity Aroma Pu-Erh Tea via GC-O-MS, GC-MS/MS, Aroma Recombination, and Omission Tests
Source: Foods. 2025 Sep 17;14(18):3223. doi: 10.3390/foods14183223 (PMC12469430; doi:10.3390/foods14183223)
Supplement: Supplementary file 1 [file foods-14-03223-s001.zip › foods-3832310-supplementary.pdf]

Supporting Information for

Unraveling the Key Odorants in Floral-Fruity Aroma Pu-Erh Tea via GC-O-MS, GC-MS/MS, Aroma Recombination, and Omission Tests

Xianxiu Zhou[1,2], Jiajing Hu[2], Hongchun Cui[3], Jiahao Tang[2], Yongwen Jiang[2], Haibo Yuan[2], Jiahua Li[1,*], Yanqin Yang[2,*]

[1] *College of Tea Science, Yunnan Agricultural University, Kunming 650201, China*

[2] *State Key Laboratory of Tea Plant Germplasm Innovation and Resource Utilization, Tea Research Institute, Chinese Academy of Agricultural Sciences, Hangzhou 310008, China*

[3] *Tea Research Institute, Hangzhou Academy of Agriculture, Hangzhou 310024, China*

*Corresponding author.

E-mail addresses: 15087172337@163.com; yangyq@tricaas.com.

Table S1 The standard information of volatile compounds used in this study.

Standards	Related information			
	Purity	Brand	City	Country
Citral	>98%	Yuanye	Shanghai	China
1-Nonanol	99.50%	Yingxin	Shanghai	China
(E, E)-2,4-Heptadienal	≥90%	TRC	Toronto	Canada
Hexanal	>98%	TCI	Shanghai	China
1-Hexanol	98%	TCI	Shanghai	China
Heptanal	≥99.5%	TCI	Shanghai	China
1-Heptanol	98%	TCI	Shanghai	China
Nonanal	>95%	TCI	Shanghai	China
2,4-Dimethyl-1-heptene	≥98%	Meryer	Shanghai	China
Geranyl acetone	97%	Meryer	Shanghai	China
1-Penten-3-ol	98%	Macklin	Shanghai	China
(E)-2-Hexenol	97%	Macklin	Shanghai	China
Benzaldehyde	98%	Macklin	Shanghai	China
3-Octanone	98%	Macklin	Shanghai	China
D-Limonene	99%	Macklin	Shanghai	China
Benzyl alcohol	99.50%	Macklin	Shanghai	China
3-Nonen-2-one	≥96%	Macklin	Shanghai	China
(E, Z)-2,6-Nonadienal	≥95%	Macklin	Shanghai	China
(E)-2-Nonenal	>95%	Macklin	Shanghai	China
Methyl salicylate	>99%	Macklin	Shanghai	China
(E, E)-2,4-Nonadienal	>90%	Macklin	Shanghai	China
Indole	>99.5%	Macklin	Shanghai	China
(E, E)-2,4-Decadienal	>90%	Macklin	Shanghai	China
β-Damascone	98%	Macklin	Shanghai	China
cis-Jasmone	98%	Macklin	Shanghai	China
Linalool	98%	J&K	Shanghai	China
Phenylethyl alcohol	99.5%	J&K	Shanghai	China
Decanal	97%	J&K	Shanghai	China
6-Methyl-5-heptene-2-one	98%	Alfa Aesar	Shanghai	China
1-Octen-3-ol	98%	Aladdin	Shanghai	China
Phenylacetaldehyde	99%	Aladdin	Shanghai	China
Guaiacol	98%	Aladdin	Shanghai	China
Geraniol	≥98%	Aladdin	Shanghai	China
Nerolidol	≥98%	Aladdin	Shanghai	China
β-Ionone	>95%	TCI	Shanghai	China
2-Methylpropanal	>98%	TCI	Shanghai	China
3-Methylbutanal	>98%	TCI	Shanghai	China
Nerol	>98%	Aladdin	Shanghai	China
Theaspirane	≥90%	Macklin	Shanghai	China

(Z)-Hexanoic acid, 3-hexenyl ester	98%	Macklin	Shanghai	China
2,2,6-Trimethyl-cyclohexanone	≥96%	Yuanye	Shanghai	China
Dihydroactinidiolide	≥99%	Medlife	Shanghai	China
Pentanal	≥95%	J&K	Shanghai	China

Table S2 The information of calibration curves.

Compounds	Qualitative ion pairs	Quantitative ion pairs	Calibration curves	R^2
1-Pentene-3-ol	72→42	72→57	y=89.9389x+959.533	0.9995
Isovaleraldehyde	71→43	71→41	y=593834x+7609.9453	0.9799
Isobutyraldehyde	72→43	72→57	y=884.388x+10483.3949	0.9988
Hexanal	72→44	72→43	y=359.1220x+18342.7010	0.9879
(E)-2-Hexenol	91→61	91→65	y=124946.329719x+1135303.686499	0.9984
Benzaldehyde	77→74	77→50	y=25619.1338x-15854.4924	0.9966
Heptanol	70→42	70→55	y=30107.7738x+97379.1675	0.9966
6-Methyl-5-hepten-2-one	108.1→93	108.1→79	y=1323.6369x+288.7823	0.9905
1-Octen-3-ol	99→71	99→43	y=9349.845001x+108.1975	0.9918
(*E*, *E*)-2,4-Heptadieenal	108.1→93	108.1→79	y=62342.7839x-186221.714352	0.9942
D-Limonene	93→77	93→91.2	y=686485.224818x+41058.2056	0.9920
3-Octanone	108.1→90.8	108.1→92.9	y=25959.0513849x+1428721.8465	0.9929
Benzyl alcohol	108→93	108→80	y=15789.313700x-208.87	0.9951
2,2,6-Trimethyl-cyclohexanone	140→99	140→82	y=17072.546078x+71615.301757	0.9997
Eugenol	109→80	109→78	y=692.472589x+1647.855262	0.9979
Linalool	93→77	93→91.2	y=135172.429501x+4435.1.441362	0.9980
Phenylethyl alcohol	122→97	122→92	y=1784.804523x-19447.349458	0.9988
3-Nonene-2-one	125→92	125→94	y=166.054131x-67.165896	0.9995
(E, Z)-2,6-Nonadienal	70→55	70→42	y=38443.435250x+104388.169443	0.9989
Nonanal	98→56	98→69	y=40.614073x+2917.349031	0.9925
1-Nonanol	108.1→93	108.1→79	y=52887.965451x-176590.684787	0.9934
Methyl salicylate	152→136	152→121	y=5566.713322x-5465.09037	0.9984
Decanal	112→69	112→70	y=2102.800125x+5739.950530	0.9966

(*E*)-2-Nonenal	83→41	83→55	y=63170.483015x+213513.485789	0.9948
(*E*, *E*)-2,4-Nonadienal	81→79	81→53	y=75381.831109x+428638.068158	0.9996
Neroleol	93→91	93→77	y=6005.367594x+4635.813866	0.9935
Citral	84→41	84→56	y=9639.066785x+56418.992161	0.9973
Geraniol	93→77	93→91	y=99792.495741x-99199.860325	0.9946
Theaspirane	138→109	138→96	y=81257.761615x+121346.623114	0.9998
Indole	117→115	117→90	y=146772.159835x-189843.532110	0.9931
(*E*,*E*)-2,4-Decadienal	81→79	81→53	y=188910.915724x-551521.354313	0.9962
cis-3-Hexenyl caproate	99→71	99→57	y=26307.948250x-12448.429776	0.9986
β- Damastone	123→89	123→95	y=6585.011844x+2778.666626	0.9981
cis-Jasmine	164→150	164→135	y=2719.322142x+8182.617305	0.9928
Geranil acetone	136→108	136→121	y=15771.9019949x-1529.553778	0.9998
Nerolidol	93→77	93→91	y=145097.590576x+239590.561300	0.9996
β-Ionone	177→107	177→121	y=745.083629x+297.534256	0.9996

Table S3 The information of identified volatile compounds in SAPET and FFAPET samples.

No.	Compounds	RT[a]	RI[b]	RI[c]	Contents		Identification methods	*P*	VIP
					SAPET	FFAPET			
Aldehydes									
1	2-Methylbutanal	2.78	704	682	6.82±2.8	1.38±1.22	MS, RI	<0.001	1.21
2	Pentanal	3.25	716	717	3.92±1.85	0.39±0.36	MS, RI	0.82	0.34
3	Crotonaldehyde	4.20	740	744	0.96±0.3	0.16±0.14	MS, RI	<0.001	1.74
4	(*E*)-2-Pentenal	4.56	749	746	1.78±1.14	0.35±0.43	MS, RI	0.074	0.59
5	Hexanal	6.09	787	788	1.86±0.16	1.79±0.09	MS, RI, Std	0.792	0.22

6	3-Furaldehyde	7.24	815	815	5.91±6.32	1.6±2.51	MS, RI	0.826	0.29
7	(*E*)-2-Hexanal	8.25	840	835	292.89±176.72	115.31±71.51	MS, RI, Std	<0.001	1.18
8	(*Z*)-4-Heptenal	10.25	890	905	2.51±0.87	0.55±0.44	MS, RI	<0.001	1.57
9	Heptanal	10.35	893	891	0.16±0.21	0.03±0.06	MS, RI, Std	0.002	0.80
10	5-Methyl-2-furanaldehyde	12.97	958	957	0.89±1.47	1.61±2.15	MS, RI	0.277	0.71
11	Benzaldehyde	12.90	956	956	551.61±153.5	254.71±127.12	MS, RI, Std	<0.001	1.46
12	Octanal	14.78	1003	1003	1.4±0.94	0.43±0.24	MS, RI	0.004	0.78
13	(*E*, *E*)-2,4-Heptadienal	15.12	1011	1007	12.36±5.28	8.88±3.46	MS, RI, Std	0.001	0.84
14	Benzeneacetaldehyde	16.40	1043	1042	5.92±3.46	6.29±5.07	MS, RI	0.971	0.76
15	(*E*)-2-Octenal	17.05	1060	1059	1.95±3.11	1.78±3.41	MS, RI	0.724	0.46
16	4-Methylbenzaldehyde	17.30	1066	1081	1.13±0.78	0.19±0.18	MS, RI	<0.001	1.25
17	(*E*)-2-Nonenal	21.06	1159	1157	4.1±0.37	5.09±1.23	MS, RI, Std	0.001	1.00
18	2,4-Dimethylbenzaldehyde	21.59	1172	1175	1.3±0.8	0.58±0.54	MS, RI	<0.001	1.17
19	Safranal	22.47	1194	1197	5.78±2.86	1.26±1.25	MS, RI	<0.001	1.42
20	Decanal	22.80	1202	1201	2.36±0	2.36±0	MS, RI, Std	0.053	0.56
21	(*E*, *E*)-2,4-Nonadienal	23.20	1213	1211	9.69±2.16	5.84±1.08	MS, RI	<0.001	1.49
22	*β*-Cyclocitral	23.20	1213	1234	4.89±2.38	0.82±0.84	MS, RI, Std	<0.001	1.48
23	Isovaleraldehyde	1.93	683	663	15.65±21.77	8.39±10.43	MS, RI, Std	0.108	0.49
24	Nonanal	20.28	1140	1121	0.58±0.05	0.59±0.22	MS, RI, Std	0.987	0.14
25	Citral	24.93	1256	1247	2.77±0.02	2.79±0.05	MS, RI, Std	0.283	0.34
26	(*E*, *E*)-2,4-Decadienal	26.70	1299	1316	5.33±1.36	9.3±11.11	MS, RI, Std	0.217	0.53
27	(*E*, *Z*)-2,6-Nonadienal	20.73	1151	1155	4.17±0.22	4.31±0.21	MS, RI	0.099	0.44
Acids									
1	Propionic acid	3.53	723	725	0.64±1.22	0.21±0.23	MS, RI	0.302	0.49
2	Nonanoic acid	26.29	1289	1271	100±33.91	17.1±16.57	MS, RI	<0.001	1.56
Ketones									

1	2-Pentanone	3.06	711	700	1.27±0.43	0.14±0.14	MS, RI	<0.001	1.50
2	3-Pentene-2-one	4.17	739	739	0.25±0.22	0.1±0.13	MS, RI	0.001	0.90
3	3-Hexanone	4.39	744	746	1.75±2.61	0.2±0.2	MS, RI	0.005	0.72
4	2-Hexanone	5.65	776	778	1.18±1.14	0.1±0.06	MS, RI	<0.001	1.08
5	2-Heptanone	9.81	879	880	8.81±6.71	0.68±0.53	MS, RI	<0.001	1.32
6	2-Methyl-6-heptanone	12.64	950	949	0.74±0.51	0.08±0.04	MS, RI	<0.001	1.33
7	6-Methyl-5-heptene-2-one	13.98	983	983	6.31±3.69	1.07±0.44	MS, RI	<0.001	1.40
8	2-Octanone	14.20	988	991	2.22±1.59	1.36±1.22	MS, RI	0.853	0.65
9	2,6,6-Trimethylcyclohexanone	16.01	1034	1047	5.24±0.02	5.27±0.18	MS, RI,Std	0.621	0.15
10	3-Octen-2-one	16.07	1035	1036	0.83±0.53	0.39±0.5	MS, RI	0.005	0.81
11	Isophorone	17.00	1058	1080	12.21±6.93	1.8±1.99	MS, RI	<0.001	1.37
12	3,5-Octadienone	17.52	1071	1069	9.25±5.57	3.43±2.68	MS, RI	<0.001	1.19
13	2-Nonanone	18.38	1092	1093	1.37±1.21	0.23±0.14	MS, RI	0.002	0.96
14	6-Methyl-3,5-heptadien-2-one	18.93	1106	1107	3.27±2.99	0.61±0.82	MS, RI	<0.001	1.17
15	Maltol	19.40	1118	1139	1.38±0.49	4.31±9.3	MS, RI	0.16	0.61
16	(*Z*)-3-Nonen-2-one	20.25	1139	1146	142.14±49.13	94.04±84.49	MS, RI	0.002	0.83
17	4-Ketoisophorone	20.43	1143	1152	6.61±3.05	2±2.51	MS, RI	<0.001	1.28
18	Phenyl ethyl ketone	20.43	1144	1144	34.7±23.32	7.09±11.96	MS, RI	<0.001	1.21
19	2-Decanone	22.26	1189	1192	0.78±0.8	0.22±0.12	MS, RI	0.015	0.91
20	6-Undecanone	25.11	1260	1258	0.52±0.12	0.17±0.14	MS, RI	<0.001	1.36
21	3-Undecanone	25.69	1274	1268	0.07±0.03	0.08±0.2	MS, RI	0.878	0.31
22	Dihydro-*β*-ionone	30.47	1393	1405	0.87±0.43	0.28±0.31	MS, RI	<0.001	1.25
23	*β*-Ionone epoxide	32.01	1432	1428	11.13±8.21	1.96±2.32	MS, RI, Std	<0.001	1.29
24	Prenylacetone	13.50	971	986	8.68±5.45	6±3.63	MS, RI, Std	0.006	0.77
25	Jasmone	29.14	1360	1381	3.87±0.2	3.75±0.24	MS, RI, Std	0.009	0.71
26	*β*-Damascenone	30.00	1382	1390	1.55±1.12	1.88±1.02	MS, RI, Std	0.423	0.40

27	Geranylacetone	30.93	1405	1428	2.26±0.18	1.96±0.12	MS, RI, Std	<0.001	1.43
28	*β*-Ionone	35.11	1509	1491	6.46±1.24	9.13±7.44	MS, RI	0.111	0.43
Aromatic hydrocarbons									
1	1,3-Dimethylbenzene	8.85	855	855	13.1±8.88	2.77±2.66	MS, RI	<0.001	1.06
2	1,3,5-Trimethylbenzene	14.25	990	992	8.46±2.26	2.28±2.22	MS, RI	<0.001	1.23
3	1,2,4-Trimethylbenzene	15.36	1017	1026	3.9±1.28	0.85±0.8	MS, RI	0.287	0.54
4	*O*-Cymol	15.59	1023	1025	4.16±1.93	0.76±0.45	MS, RI	<0.001	1.36
5	1,2-Dimethyl-4-vinylbenzene	18.31	1091	1100	0.45±0.31	0.28±0.15	MS, RI	0.007	0.90
Alcohols									
1	1-Pentanol	4.97	759	759	5.18±5.3	0.15±0.1	MS, RI	<0.001	1.18
2	(*E*)-3-Hexenol	8.38	844	842	0.35±0.43	0.3±0.47	MS, RI	0.359	0.65
3	Cyclohexyl alcohol	9.01	859	874	0.94±0.53	0.16±0.14	MS, RI	<0.001	1.39
4	1-Heptanol	13.39	969	968.4	0.67±0.02	0.69±0.03	MS, RI, Std	0.925	0.34
5	3-Octenol	13.78	978	983	4.95±1.06	3.92±1.24	MS, RI, Std	<0.001	0.57
6	Benzyl alcohol	16.14	1037	1033	1.01±0.14	0.89±0.19	MS, RI, Std	0.008	0.69
7	1-Octanol	17.75	1077	1071	1.55±2.33	1.61±2.68	MS, RI	0.631	0.42
8	Linanool	18.73	1101	1100	3.68±4.28	32.37±44.84	MS, RI, Std	0.006	0.88
9	Phenylethyl alcohol	19.39	1118	1117	14.9±13.68	211.96±202.15	MS, RI, Std	<0.001	1.14
10	(*E*)-Linallol oxide (pyran)	21.60	1173	1171	11.24±13.15	14.7±13.99	MS, RI	0.048	0.97
11	*α*-Terpineole	22.36	1192	1189	31.29±16.86	34.83±32.49	MS, RI	0.25	0.94
12	Geraniol	24.40	1242	1251	54.35±45.92	88.9±84.71	MS, RI, Std	0.184	0.61
13	Penten-3-ol	1.92	683	701	4.77±0	4.77±0	MS, RI, Std	0.108	0.49
14	1-Nonanol	21.59	1172	1173	3.44±0.18	3.67±0.45	MS, RI, Std	0.076	0.71
15	Nerol	24.35	1241	1232	10.83±2.47	29.56±18.38	MS, RI, Std	<0.001	1.07
16	Nerolidol	34.41	1491	1512	6.17±0.38	5.93±0.35	MS, RI, Std	0.004	0.79

Alkanes									
1	4-Methyldecane	17.14	1062	1060	2.5±0.45	0.63±0.49	MS, RI	0.048	0.57
2	3-Methylundecane	21.48	1170	1171	4.32±1	1.75±1.19	MS, RI	<0.001	1.32
Alkenes									
1	1-Ethyl-5,5-dimethylcyclopenta-1,3-diene	7.76	828	856	1.22±1.03	0.14±0.12	MS, RI	<0.001	1.15
2	3,5,5-Trimethyl-2-hexene	13.39	968	968	4.49±1.62	21.7±24.23	MS, RI	0.015	0.66
3	*α*-Tetradecene	29.31	1364	1388	2.15±2.52	3.84±2.36	MS, RI	0.006	0.78
4	Thujopsene	30.61	1397	1410	0.44±0.29	0.15±0.17	MS, RI	<0.001	1.06
5	*β*-Elemene	29.31	1364	1366	0.52±0.14	0.16±0.15	MS, RI	<0.001	1.21
6	2,4-Dimethyl-1-heptene	7.68	826	855	0.64±0	0.65±0.01	MS, RI	0.002	0.79
7	*D*-Limonene	15.77	1028	1028	2.12±0.7	1.55±0.96	MS, RI, Std	0.007	0.70
Heterocyclic compounds									
1	α-Ethylpyridine	10.30	892	910	22.82±12.47	3.45±5.11	MS, RI	<0.001	1.43
2	1-Ethyl-2-formylpyrrole	16.54	1047	1046	40.19±38.19	58.86±54.82	MS, RI	0.05	0.94
3	1H-Pyrrole	18.18	1088	1072	4.08±3.41	0.89±1.27	MS, RI	0.003	0.79
4	2-Ethylfuran	3.26	716	720	3.72±1.33	0.63±0.66	MS, RI	<0.001	1.47
5	2-Butylfuran	9.84	880	878	0.62±0.27	0.09±0.06	MS, RI	<0.001	1.57
6	Ethylpyrazine	10.31	892	893	9.04±6.56	0.8±1.9	MS, RI	<0.001	1.22
7	2-Pentylfuran	14.18	988	988	5.57±3.68	0.77±0.54	MS, RI	<0.001	1.34
8	(*Z*)-2-(2-Pentenyl)furan	14.55	997	1001	0.26±0.16	0.07±0.05	MS, RI	<0.001	1.24
9	2,5-Furandicarbaldehyde	16.54	1047	1034	39.16±38.34	58.75±54.69	MS, RI	0.05	0.92
10	2-Methyl-5-acetylfuran	16.00	1033	1040	0.35±0.32	0.23±0.21	MS, RI	0.197	1.04
11	3-Phenylfuran	23.23	1213	1225	0.03±0.02	0.06±0.09	MS, RI	0.113	0.67
12	2-Cyano-6-methoxybenzothiazole	23.33	1216	1225	0.07±0.18	0.01±0.01	MS, RI	0.156	0.38
13	Indole	25.69	1274	1289	10.58±2.15	7.95±1.75	MS, RI, Std	<0.001	1.44

14	Theaspirane	26.02	1283	1298	0.99±0.04	0.97±0.01	MS, RI, Std	0.001	0.87
Esters									
1	Methyl valerate	6.66	801	806	0.08±0.09	0.03±0.04	MS, RI	0.01	0.66
2	*γ*-Butyrolactone	10.36	893	891	1.27±3.13	0.15±0.36	MS, RI	0.093	0.44
3	Amyl acetic ester	10.35	893	892	5.78±2.87	0.58±0.45	MS, RI	<0.001	1.49
4	Methyl hexoate	11.36	918	914	0.2±0.22	0.05±0.08	MS, RI	0.001	0.96
5	Methyl enanthate	15.82	1029	1021	1.56±0.21	0.45±0.52	MS, RI	<0.001	1.28
6	Methyl caprylate	20.25	1139	1123	7.62±3.7	1.45±1.39	MS, RI	<0.001	1.32
7	Methyl benzeneacetate	21.15	1162	1164	0.65±1.12	6.81±7.72	MS, RI	0.001	1.04
8	Salicylic acid, methyl ester	22.36	1192	1190	4.19±0.41	5.76±3.98	MS, RI, Std	0.111	0.60
9	Methyl nonanoate	23.43	1218	1225	0.05±0.06	0.05±0.07	MS, RI	0.446	0.34
10	Phenethyl acetate	24.50	1245	1243	0.12±0.16	1.31±1.18	MS, RI	<0.001	1.18
11	*γ*-Octalactone	25.37	1267	1262	0.09±0.03	0.07±0.07	MS, RI	0.418	0.65
12	(*E*)-Geranic acid methyl ester	26.65	1298	1315	0.13±0.05	0.6±0.52	MS, RI	<0.001	1.16
13	*α*-Terpenyl acetate	27.53	1320	1340	0.12±0.13	0.03±0.02	MS, RI	<0.001	0.90
14	*γ*-Nonalactone	28.11	1335	1335	1.01±1.23	0.59±0.37	MS, RI	0.048	0.92
15	(*Z*)-3-Hexenyl hexanoate	28.70	1349	1375	0.1±0	0.1±0	MS, RI, Std	0.012	0.69
16	Dihydroactinidiolide	33.39	1466	1492	1.29±0.48	1.34±0.72	MS, RI, Std	0.638	0.26
Methoxy-phenolic compounds									
1	*m*-Dimethoxybenzene	20.44	1144	1143	120.61±69.74	44.39±45.05	MS, RI	<0.001	1.12
2	Guaiacol	18.01	1083	1096	21.29±7.17	15.43±6.61	MS, RI, Std	<0.001	0.93

Note: RT[a] represented retention time; RI[b] represented the retention indices calculated from a series of n-alkanes (C7-C40); RI[c] represented retention indices referred to the literature values with HP-5ms column or equivalent chromatographic column [NIST Chemistry WebBook (http://webbook.nist.gov/chemistry/) and http://www.flavornet.org/flavornet.html/]. MS represented identification based on the NIST 11 database; Std represented standard certification. FFAPET represented floral-fruity aroma Pu-erh tea; SAPET represented stale aroma Pu-erh tea.

Table S4 The odor thresholds of volatile compounds.

No.	Compounds	RT[a]	Odor threshold in water[θ] (μg/L)
Aldehydes			
1	2-Methylbutanal	2.78	1.5[a]
2	Pentanal	3.25	12[a]
3	Crotonaldehyde	4.20	--
4	(*E*)-2-Pentenal	4.56	310[a]
5	Hexanal	6.09	2.4[a]
6	3-Furaldehyde	7.24	--
7	(*E*)-2-Hexanal	8.25	30[b]
8	(*Z*)-4-Heptenal	10.25	0.0087[a]
9	Heptanal	10.35	2.8[b]
10	5-Methyl-2-furanaldehyde	12.97	--
11	Benzaldehyde	12.90	3[b]
12	Octanal	14.78	0.7[b]
13	(*E*, *E*)-2,4-Heptadienal	15.12	0.032[a]
14	Benzeneacetaldehyde	16.40	0.04[b]
15	(*E*)-2-Octenal	17.05	1.7[a]
16	4-Methylbenzaldehyde	17.30	--
17	(*E*)-2-Nonenal	21.06	0.08[b]
18	2,4-Dimethylbenzaldehyde	21.59	--
19	Safranal	22.47	0.7[b]
20	Decanal	22.80	2.6[c]
21	(*E*, *E*)-2,4-Nonadienal	23.20	0.046[b]
22	*β*-Cyclocitral	23.20	3[b]

23	Isovaleraldehyde	1.93	0.5[a]
24	Nonanal	20.28	1[a]
25	Citral	24.93	0.00015[a]
26	(*E*, *E*)-2,4-Decadienal	26.70	0.07[b]
27	(*E*, *Z*)-2,6-Nonadienal	20.73	0.0045[b]
Acids			
1	Propionic acid	3.53	16000[a]
2	Nonanoic acid	26.29	26[a]
Ketones			
1	2-Pentanone	3.06	--
2	3-Pentene-2-one	4.17	--
3	3-Hexanone	4.39	--
4	2-Hexanone	5.65	--
5	2-Heptanone	9.81	3000[a]
6	2-Methyl-6-heptanone	12.64	--
7	6-Methyl-5-heptene-2-one	13.98	50[c]
8	2-Octanone	14.20	--
9	2,6,6-Trimethylcyclohexanone	16.01	0.1[b]
10	3-Octen-2-one	16.07	--
11	Isophorone	17.00	11[b]
12	3,5-Octadienone	17.52	100[b]
13	2-Nonanone	18.38	0.5[a]
14	6-Methyl-3,5-heptadien-2-one	18.93	--
15	Maltol	19.40	5000[a]
16	(*Z*)-3-Nonen-2-one	20.25	800[b]
17	4-Ketoisophorone	20.43	25[a]

18	Phenyl ethyl ketone	20.43	65[d]
19	2-Decanone	22.26	--
20	6-Undecanone	25.11	--
21	3-Undecanone	25.69	--
22	Dihydro-*β*-ionone	30.47	0.001[b]
23	*β*-Ionone epoxide	32.01	--
24	Prenylacetone	13.50	0.16[a]
25	Jasmone	29.14	0.26[a]
26	*β*-Damascenone	30.00	0.0013[b]
27	Geranylacetone	30.93	60[b]
28	*β*-Ionone	35.11	0.021[a]
Aromatic hydrocarbons			
1	1,3-Dimethylbenzene	8.85	--
2	1,3,5-Trimethylbenzene	14.25	--
3	1,2,4-Trimethylbenzene	15.36	--
4	*O*-Cymol	15.59	--
5	1,2-Dimethyl-4-vinylbenzene	18.31	--
Alcohols			
1	1-Pentanol	4.97	--
2	(*E*)-3-Hexenol	8.38	3.9[a]
3	Cyclohexyl alcohol	9.01	470[d]
4	1-Heptanol	13.39	400[c]
5	3-Octenol	13.78	1[b]
6	Benzyl alcohol	16.14	100[b]
7	1-Octanol	17.75	3[c]
8	Linalool	18.73	0.22[b]

9	Phenylethyl alcohol	19.39	0.35[c]
10	(*E*)-Linalool oxide (pyranoid))	21.60	6[a]
11	*α*-Terpineole	22.36	330[b]
12	Geraniol	24.40	3.2[a]
13	Penten-3-ol	1.92	400[c]
14	1-Nonanol	21.59	1[a]
15	Nerol	24.35	0.3[b]
16	Nerolidol	34.41	10[b]
Alkanes			
1	4-Methyldecane	17.14	--
2	3-Methylundecane	21.48	--
Alkenes			
1	1-Ethyl-5,5-dimethylcyclopenta-1,3-diene	7.76	--
2	3,5,5-Trimethyl-2-hexene	13.39	--
3	*α*-Tetradecene	29.31	--
4	Thujopsene	30.61	--
5	*β*-Elemene	29.31	--
6	2,4-Dimethyl-1-heptene	7.68	--
7	*D*-Limonene	15.77	1.2[b]
Heterocyclic compounds			
1	*α*-Ethylpyridine	10.30	17[a]
2	1-Ethyl-2-formylpyrrole	16.54	--
3	1H-Pyrrole	18.18	--
4	2-Ethylfuran	3.26	17[a]

5	2-Butylfuran	9.84	--
6	Ethylpyrazine	10.31	--
7	2-Pentylfuran	14.18	4.8[a]
8	(*Z*)-2-(2-Pentenyl) furan	14.55	--
9	2,5-Furandicarbaldehyde	16.54	--
10	2-Methyl-5-acetylfuran	16.00	--
11	3-Phenylfuran	23.23	--
12	2-Cyano-6-methoxybenzothiazole	23.33	--
13	Indole	25.69	500[b]
14	Theaspirane	26.02	0.0002[b]
Esters			
1	Methyl valerate	6.66	--
2	γ-Butyrolactone	10.36	--
3	Amyl acetic ester	10.35	--
4	Methyl hexoate	11.36	70[c]
5	Methyl enanthate	15.82	--
6	Methyl caprylate	20.25	--
7	Methyl benzeneacetate	21.15	--
8	Methyl salicylate	22.36	40[b]
8	Methyl nonanoate	23.43	--
10	2-Phenylethyl acetate	24.50	0.02[a]
11	γ-Octalactone	25.37	6.5[a]
12	(*E*)-Geranic acid methyl ester	26.65	--
13	α-Terpenyl acetate	27.53	--
14	γ-Nonalactone	28.11	9.7[b]
15	(*Z*)-3-Hexenyl hexanoate	28.70	16[b]

16	Dihydroactinidiolide	33.39	500[a]
Methoxy-phenolic compounds			
1	*m*-Dimethoxybenzene	20.44	--
2	Guaiacol	18.01	--

Note: "θ, the odor thresholds of all volatile components found in the literature: a: [1]; b [2]; c: [3]; d: [4]. "--" represented has not been found in the literature

[1] Zhai X, Zhang L, Granvogl M, Ho CT, Wan X. 2022. Flavor of tea (Camellia sinensis): A review on odorants and analytical techniques. *Comprehensive Review in Food Science and Foods safety* 21:3867-909.

[2] Chen, G., Zhu, G., Xie, H., Zhang, J., et al.2024. Characterization of the key differential aroma compounds in five dark teas from different geographical regions integrating GC–MS, ROAV and chemometrics approaches. *Food Research International, 194*, 114928.

[3] Xie J, Wang L, Deng Y, Yuan H, Zhu J, et al. 2023. Characterization of the key odorants in floral aroma green tea based on GC-E-Nose, GC-IMS, GC-MS and aroma recombination and investigation of the dynamic changes and aroma formation during processing. *Food Chemistry* 427:136641.

[4] Guo X, Ho CT, Wan X, Zhu H, Liu Q, Wen Z. 2021. Changes of volatile compounds and odor profiles in Wuyi rock tea during processing. *Food Chemistry* 341:128230.

Table S5 A total of 34 volatile components with OAV＞1 in FFAPET samples.

No.	Compounds	Aroma attributes	OAV range	Number of samples with OAV>1
1	Theaspirane	Woody[b]	4810.06—4932.17	10
2	(*E*, *Z*)-2,6-Nonadienal	Green[b]	905.54—1007.83	10
3	*β*-Damascenone	Floral[b]	417.71—2607.62	10
4	*β*-Ionone	Floral, fruity[a]	287.79—1153.57	10
5	Dihydro-*β*-ionone	Floral[b]	31.6—938.53	10
6	Phenylethyl alcohol	Floral, fruity[c]	61.8—1942.45	10
7	(*E*, *E*)-2,4-Heptadienal	Fatty[a]	147.87—478.78	10
8	Benzeneacetaldehyde	Green[b]	49.05—285	10
9	(*E*, *E*)-2,4-Nonadienal	Fatty[b]	74.13—160.09	10
10	(*Z*)-4-Heptenal	Fatty[a]	18.42—178.89	10
11	2,6,6-Trimethylcyclohexanone	Honey-like, honeylike[b]	52.07—54.92	10
12	(*E*)-2-Nonenal	Cucumber-like[b]	50.69—97.74	10
13	(*E*, *E*)-2,4-Decadienal	Fatty[b]	36.19—464.23	10
14	Nerol	Floral, fruity[b]	26.29—215.35	10
15	Isovaleraldehyde	Malty[a]	6.51—46.28	10
16	Benzaldehyde	Fruity, Almond-like[b]	18.4—125.65	10
17	Linalool	Floral, fruity[b]	0—633.59	9
18	Jasmone	Floral, fruity[a]	13.51—15.42	10
19	2-Phenylethyl acetate	Rose, honey[a]	3.07—158.42	10
20	(*E*)-2-Hexanal	Green[b]	2.08—8.63	10
21	Prenylacetone	Fruity[a]	6.51—63.76	10
22	Geraniol	Floral[a]	6.24—86.43	10
23	(*E*)-Linalool oxide (pyranoid))	Floral, fruity[a]	0.68—7.69	7
24	2-Methylbutanal	Malty[a]	0.28—4.78	5

25	1-Nonanol	Floral[a]	3.3—4.67	10
26	3-Octenol	Mushroom-like[b]	2.9—6.49	10
27	2-Nonanone	Green[a]	0.21—3.5	2
28	Octanal	Fruity[b]	0.25—2.47	2
29	Safranal	Woody[b]	0.55—5.78	8
30	Pentanal	Almond-like, malty[a]	0.01—2.14	2
31	Nonanoic acid	Musty[a]	0.07—2.4	4
32	*D*-Limonene	Fruity[b]	0.97—2.42	8
33	(*E*)-2-Octenal	Floral, ruity[a]	0.06—5.03	2
34	1-Octanol	Matallic[c]	0.03—2.36	3

Note: "θ", the aroma attributes of all volatile components found in the literature: a: [1]; b [2]; c: [3]; d: [4]. FFAPET represented floral-fruity aroma Pu-erh tea.

[1] Zhai X, Zhang L, Granvogl M, Ho CT, Wan X. 2022. Flavor of tea (Camellia sinensis): A review on odorants and analytical techniques. *Comprehensive Review in Food Science and Foods safety* 21:3867-909.

[2] Chen, G., Zhu, G., Xie, H., Zhang, J., et al.2024. Characterization of the key differential aroma compounds in five dark teas from different geographical regions integrating GC–MS, ROAV and chemometrics approaches. *Food Research International, 194*, 114928.

[3] Xie J, Wang L, Deng Y, Yuan H, Zhu J, et al. 2023. Characterization of the key odorants in floral aroma green tea based on GC-E-Nose, GC-IMS, GC-MS and aroma recombination and investigation of the dynamic changes and aroma formation during processing. *Food Chemistry* 427:136641.

[4] Guo X, Ho CT, Wan X, Zhu H, Liu Q, Wen Z. 2021. Changes of volatile compounds and odor profiles in Wuyi rock tea during processing. *Food Chemistry* 341:128230.

Table S6 The information of volatile compounds in representative FFAPET2 identified by GC-O-MS.

NO.	Compounds	RT[a]	RI[b]	Class	Odor descriptions	FD
1	1-Hexanol	1370	1387	Alcohols	Sweet	4
2	Linalool oxide 1	1462	1450	Alcohols	Floral	1
3	2-Ethylhexanol	1499	1490	Alcohols	Mushroom	1
4	Linalool	1554	1551	Alcohols	Floral, rose	128
5	Furfuryl alcohol	1667	1678	Alcohols	Caramel, bread	1
6	Geraniol	1847	1857	Alcohols	Floral, rose	128
7	Benzyl alcohol	1884	1898	Alcohols	Fruity, mild sweet	1
8	Phenylethyl alcohol	1919	1935	Alcohols	Fruity, honey-like, citrus	128
9	Nerolidol	2034	2042	Alcohols	Floral, fruity	4
10	*O*-Methylisoeugenol	2171	2185	Methoxy-phenolic compounds	Sweet	2
11	Methyleugenol	2010	2030	Methoxy-phenolic compounds	Clove, spicy	1
12	1,2,3-Trimethoxy-5-methylbenzene	2042	2041	Methoxy-phenolic compounds	Stale	1
13	1,2,4-Trimethoxybenzene	2082	2094	Methoxy-phenolic compounds	Stale, musty, herbal	1
14	Hexanal	1081	1081	Aldehydes	Green, grassy, leafy vegetable	2
15	(*Z*)-4-Heptenal	1265	1238	Aldehydes	Fatty	128
16	Octanal	1314	1296	Aldehydes	Pungent, spicy	1
17	Nonanal	1412	1396	Aldehydes	Fruity, floral, rose	4
18	(*E*)-2-Octenal	1453	1428	Aldehydes	Green	4
19	(*E*, *E*)-2,4-Heptadienal	1517	1497	Aldehydes	Fatty	1
20	(*E, Z*)-2,6-Nonadienal	1604	1589	Aldehydes	Fatty	64
21	Perillaldehyde	1752	1759	Aldehydes	Fruity	1
22	2,2,6-Trimethylcyclohexanone	1348	1336	Ketones	Sweet, honey-like	8
23	3-Methyl-4-heptanone	1399	1403	Ketones	Fruity	1

24	Acetophenone	1676	1667	Ketones	Rancid, sour	1
25	Furan, 2-pentanoyl	1718	1747	Ketones	Sweet	1
26	2-Methylacetophenone	1730	1738	Ketones	Sweet, fruity	32
27	*β*-Ionone	1947	1957	Ketones	Floral, violet-like	8
28	*D*-Limonene	1211	1212	Alkenes	Fruity	1
29	Lavender lactone	1689	1679	Esters	Fruity	16
30	2-Phenylethyl acetate	1829	1835	Esters	Floral	32
31	Methyl hexadecanoate	2191	2208	Esters	Floral	4

Note: RT[a] represented the retention indices calculated from a series of *n*-alkanes (C7-C40); RI[b] represented retention indices referred to the literature values with HP-Innowax column or equivalent chromatographic columns [NIST Chemistry WebBook (http://webbook.nist.gov/chemistry/) and http://www.flavornet.org/flavornet.html/]. FFAPET represented floral-fruity aroma Pu-erh tea.

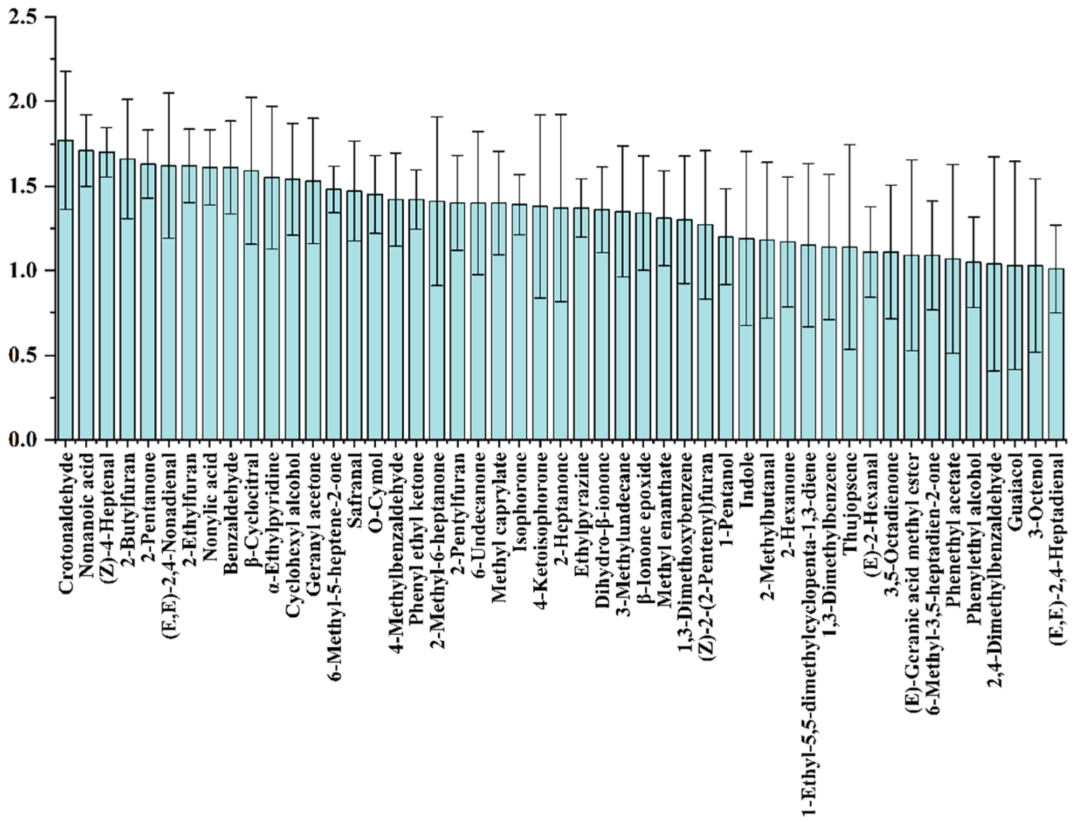

Figure S1. Key differential volatile compounds with VIP >1.
